# Case Report: An azygos vein aneurysm with dysphagia assisted diagnosis by esophageal endoscopic ultrasonography

**DOI:** 10.3389/fonc.2025.1516064

**Published:** 2025-08-22

**Authors:** Xueyan Guo, Yuanhang Xu, Yanfei Cao, Yan Jin

**Affiliations:** ^1^ Department of Gastroenterology, Shaanxi Provincial People’s Hospital, Xi’an, China; ^2^ Department of Thoracic Surgery, Shaanxi Provincial People’s Hospital, Xi’an, China

**Keywords:** dysphagia, AVA, mediastinal mass, cystic lesion, EUS

## Abstract

**Background:**

Azygos vein aneurysm (AVA) is a rare thoracic pathology that is frequently misdiagnosed. While contrast-enhanced chest computed tomography (CT) or magnetic resonance imaging (MRI) can delineate AVA location and size, these techniques lack the capability for dynamic real-time assessment of internal architecture.

**Case presentation:**

We present a highly unusual case of a 67-year-old woman who had an isolated azygos vein aneurysm presenting with dysphagia. The mass was first found by a chest X-ray. Subsequent contrast-enhanced chest CT revealed a 3.5 × 3.74 × 1.4 cm soft-tissue mass in the posterior mediastinum, suggestive of a lymph node. In contrast, esophageal endoscopic ultrasonography (EUS) demonstrated intact esophageal mucosa with extrinsic stenosis. The dynamic observation through EUS displayed that the mass was not a lymph node but a solitary cystic lesion with no internal blood flow, and there was no communication between the posterior azygos vein and the aorta. We considered that the dysphagia was caused by the cystic lesion. Thoracoscopic surgery was finally performed, which confirmed the mass as an AVA through pathological analysis.

**Conclusions:**

EUS is one of the most effective and vital tools for the preoperative evaluation of AVA.

## Introduction

Azygos vein aneurysm (AVA) is an extremely rare angiogenic disease of a mediastinal mass (1). Osler first reported AVA in 1915 (2), and it was later described as an idiopathic lesion by Walker in 1963 (3). When it occurs, it is difficult to diagnose accurately due to its rarity, and AVA may mimic a calcified mediastinal lymph node or a mediastinal mass such as a neurogenic tumor or a bronchogenic cyst (1, 4, 5). In most cases, AVA patients are asymptomatic. AVA can also be found by chest radiograph, chest computed tomography (CT), or magnetic resonance imaging (MRI) (6, 7). However, chest pain, shortness of breath, dysphagia, and other symptoms can occur if AVA constantly expands and compresses surrounding organs (esophagus, superior or inferior cava vein, right main bronchus, or trachea) or is associated with thrombosis (8).

**Figure 1:**
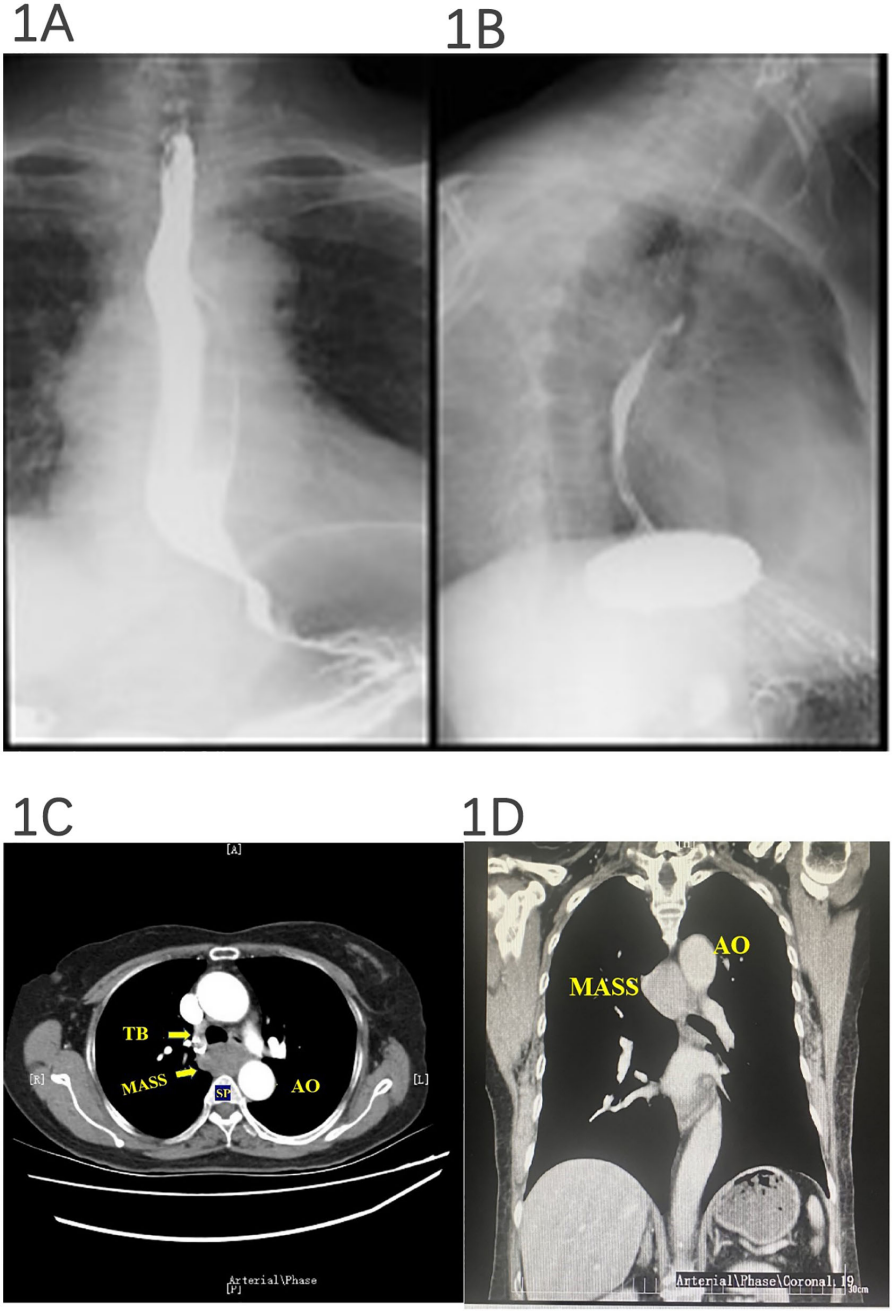
Barium meal fluoroscopy and esophageal and thoracic enhanced CT. The esophageal mucosa was smooth, barium could pass through smoothly, and peristalsis was seen, but the esophagus under the aortic arch was partially arc-shaped under pressure **(A, B)**. **(A)** Esophageal luminal compression. **(B)** Azygos vein aneurysm. A posterior mediastinal mass was detected, and a subsequent contrast CT of the thorax revealed a 3.5 × 3.74 × 1.4-cm hypodense posterior mediastinal mass without enhanced signal that was located at the level of thoracic vertebrae 4–6, behind the trachea, under the carina, and in front of the esophagus **(C, D)**.

In our case, a 67-year-old woman presented to the hospital because of progressive dysphagia for 2 months. The patient could consume liquid food but reported intermittent solid food dysphagia. First, the patient underwent barium meal fluoroscopy of the esophagus. The results showed that the esophageal mucosa was smooth, barium could pass through smoothly, and the esophagus could undergo peristalsis, but the esophagus under the aortic arch was partially arc-shaped under pressure (as demonstrated in **Figures 1A, B**). Therefore, we suspected that the patient’s dysphagia was caused by local esophageal pressure. Then, the patient underwent an enhanced thoracic CT scan. It revealed a soft-tissue mass (3.5 × 3.74 × 1.4 cm) occupying the posterior mediastinum without enhancement that was located at the level of thoracic vertebrae 4–6, behind the trachea, under the carina, and in front of the esophagus, suggesting that the mass may be a lymph node (as demonstrated in **Figures 1C, D**).

**Figure 2:**
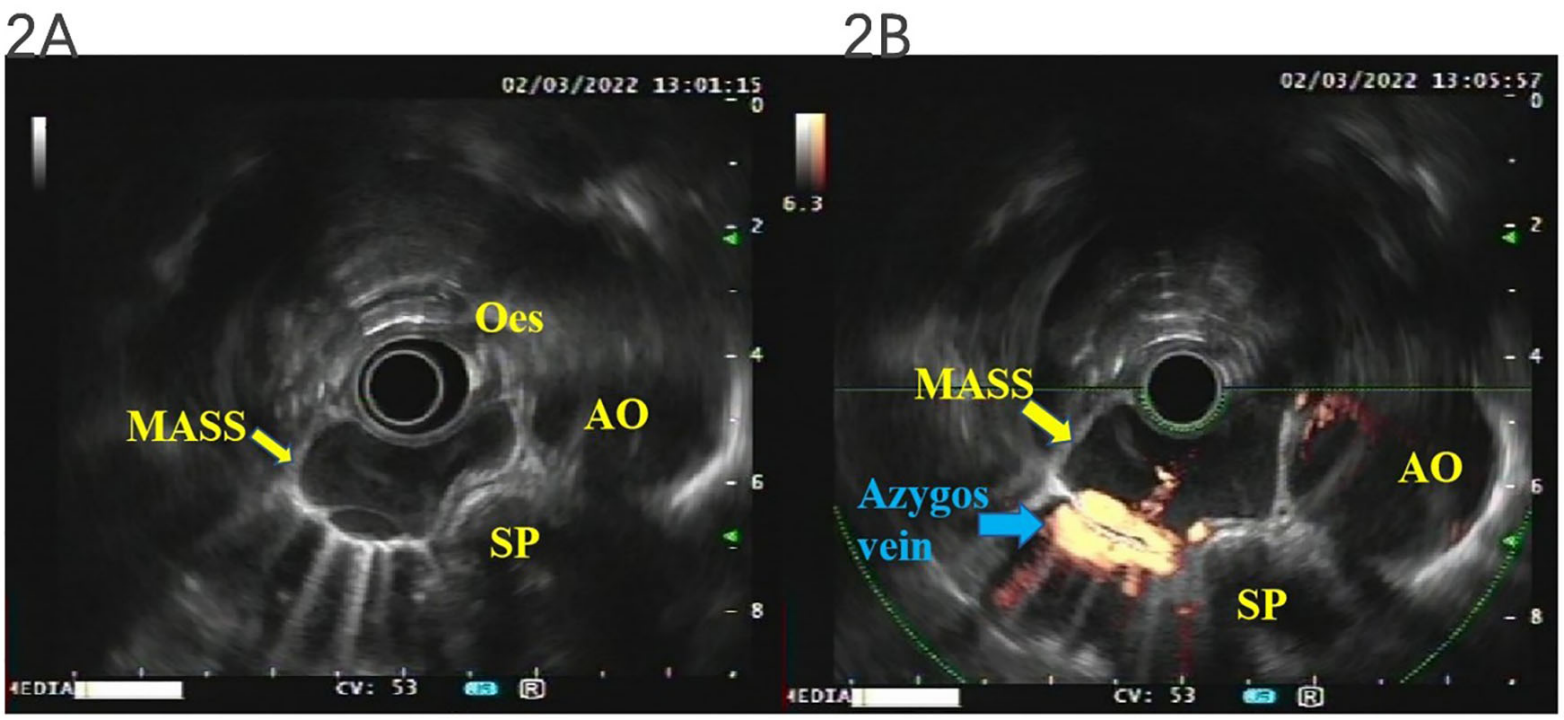
EUS. The mass was not a swollen lymph node but an idiopathic cystic tumor with no internal blood flow signal, and there was no blood flow communication between the posterior azygos vein and the thoracic aorta **(A, B)**. EUS, endoscopic ultrasonography.

Was the tumor a lymph node? What is the relationship between the mass and the esophagus? What is its internal structure? To answer these questions, we performed transesophageal endoscopic ultrasonography (EUS), which demonstrated an idiopathic cystic tumor at 25–29 cm from the incisor at the level of the middle segment of the esophagus, adjacent to the azygos vein, in front of the spine, and to the right of the aortic arch. The boundary was clear, and it could be compressed and deformed. The maximum section was 34.3 × 14.3 mm, the inner wall was smooth, no mural nodules were found, no echo could be seen inside, and an acoustic shadow could be observed. Therefore, the mass was not a swollen lymph node but an idiopathic cystic tumor with no internal blood flow signal, and there was no blood flow communication between the posterior azygos vein and the thoracic aorta (as demonstrated in **Figures 2A, B**).

**Figure 3:**
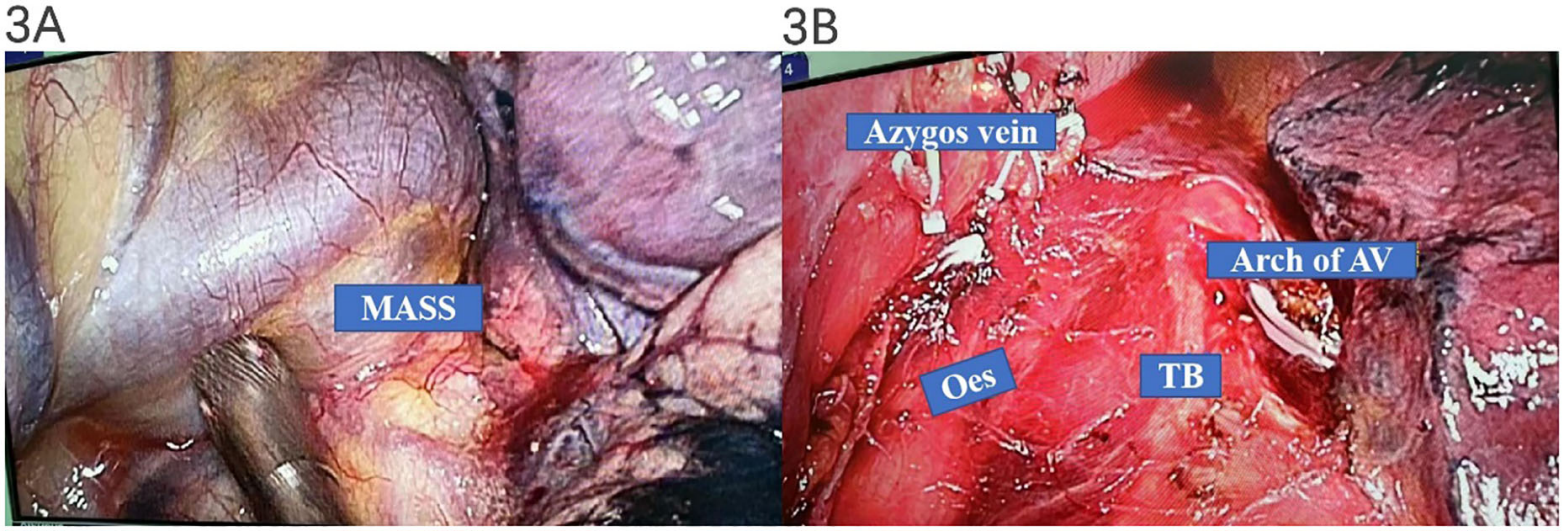
Intraoperative manifestations of thoracoscopic surgery. A 3 × 4-cm purple cystic mass with soft paste was found at the level of the azygos vein arch, which closely adhered to the azygos vein arch and had no direct relationship with the esophagus and trachea **(A, B)**.

Thoracoscopic surgery is one of the most preferred treatment options for azygos vein aneurysm (9). Thus, the mass was surgically resected via a thoracoscopic approach. During the operation, a 3 × 4-cm purple cystic mass with soft paste was found at the level of the azygos vein arch, which closely adhered to the azygos vein arch and had no direct relationship with the esophagus and trachea. The surgeon considered that the posterior mediastinal cystic mass may be an AVA (as demonstrated in **Figures 3A, B**).

**Figure 4:**
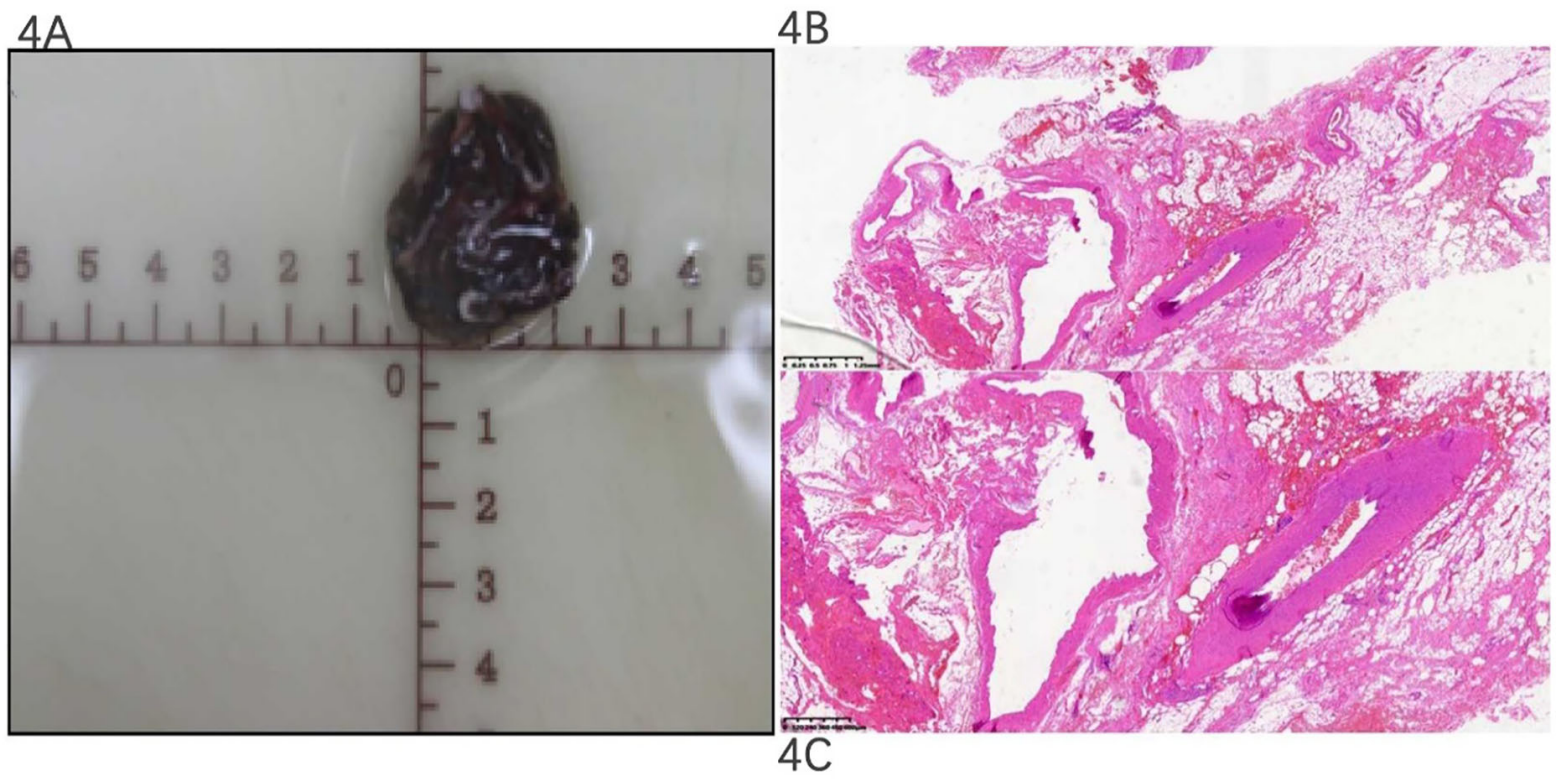
Pathological gross specimen and H&E staining section. Pathology: the mass was a grayish-red tissue with a dimension of 3.5 × 3 × 1.8 cm **(A)**. H&E staining showed that the thick wall of muscular vessels with irregular proliferation and expansion could be seen in the diseased fibrous adipose tissue, and red blood cells could be seen in the lumen, indicating an acquired saccular aneurysm of the azygos vein (B, ×,i; C, ×,xn).

After surgery, pathology showed that the mass was a grayish-red tissue, with a dimension of 3.5 × 3.0 × 1.8 cm (as demonstrated in **Figure 4A**), a polycystic disease, and a blood clot with a wall thickness of 0.2–0.3 cm. H&E staining showed that clusters of thick-walled muscular vessels with irregular proliferation and expansion could be seen in the diseased fibrous adipose tissue, and red blood cells could be seen in the lumen, indicating a venous hemangioma (as demonstrated in **Figures 4B, C**).

## Discussion

Regarding dysphagia, we first consider esophageal cancer, achalasia of the cardia, or mediastinal tumors. We rarely think of AVA because it is a rare mediastinal entity, often disguised as swollen lymph nodes, mediastinal tumors, etc. At present, we can find two reports of AVAs with dysphagia on PubMed (10, 11), which highlight that AVA is a possible cause of dysphagia.

A venous aneurysm of the extremity is defined as a persistent isolated focal venous dilatation with a diameter twofold that of the normal vein (12). AVAs have been reported for patients aged 19 to 70 years; however, the youngest patient reported was a 3-month-old infant presenting with an AVA associated with massive pulmonary embolism (13). It has been described in both male and female patients, with a higher occurrence rate in women but with no significant difference regarding patients’ age or sex (14). AVAs are categorized as saccular type or fusiform type based on CT or MRI findings. A saccular AVA is defined as an eccentric focal dilatation bulging out from a part of the azygos vein. In contrast, a fusiform AVA is defined as a circumferential short-segment spindle-shaped dilatation of the azygos vein (1). According to CT appearance, this case is a typical saccular AVA. Some studies have shown that compared with fusiform AVAs, saccular AVAs are larger and have a greater frequency of AVA-related symptoms, intralesional thromboses, and a greater than 20% short-axis growth during the follow-up period (15). Pathologically, AVA development involves congenital or acquired weakness in the venous wall architecture. Histologically, the aneurysm wall demonstrates deficient smooth muscle layers and fragmented elastic fibers (as demonstrated in **Figure 4C**), reducing tensile strength and predisposing to rupture under hemodynamic stress (16). Thrombosis within AVA arises from Virchow’s triad: blood stasis in the saccular lumen (evidenced by absent flow on EUS in our case), endothelial injury from turbulent flow, and hypercoagulability associated with local inflammation (1). Subsequent thrombus dislodgement may cause pulmonary embolism via the azygos–superior vena cava–right heart pathway, particularly in large (>3 cm) or rapidly expanding aneurysms (17).

This explains why our 3.5-cm thrombosed saccular AVA (as demonstrated in **Figure 4A**) warranted urgent intervention despite minimal symptoms. Therefore, it is particularly important for us to find the saccular AVA that caused the dysphagia.

In general, AVA is difficult to detect because it also has no clinical manifestations. If the patient has chest pain, dysphagia, cough, or other symptoms due to AVA, CT, MRI, or chest X-ray could be performed to detect a mediastinal mass, but it is difficult to accurately confirm the diagnosis. An azygos vein aneurysm shows various findings on various imaging modalities (17). It must be mentioned that dynamic MRI combined with thoracic CT scan was useful in establishing the nature of the mass and origin of vascular lesions while also identifying the vascular connections with adjacent structures (7). However, it was hard for us to detect whether there was a blood flow signal in the mass or if it was closely related to the esophagus, azygos vein, or aorta.

In this study, the patient with dysphagia underwent barium meal fluoroscopy of the esophagus, indicating that the middle part of the esophagus was compressed, but the surface mucosa of the esophagus was smooth, and the peristalsis was normal, revealing that the middle esophageal stenosis may be caused by external pressure. The most common external pressure on the middle esophagus is the external pressure of the aortic arch or mediastinal mass. Therefore, as a second step, an enhanced CT examination of the chest was performed, and a posterior mediastinal mass was found. The mass was located behind the trachea and esophagus and under the tracheal carina. While there was no enhancement in it, it was suspected that the mass may be an enlarged mediastinal lymph node.

In order to identify the precise structure involved in the mass, we found that others advocate for the use of transesophageal Doppler, which could show the presence or absence of blood flow inside the AVA (18). In the diagnostic evaluation of mediastinal lesions, ECG-gated CT angiography provides excellent visualization of macrovascular architecture and hemodynamics (e.g., azygos vein continuity). However, its spatial resolution limitation (≥0.5 mm) impedes the detailed characterization of microstructural abnormalities such as mural thrombus adhesion or venous wall layer disruption (19).

Similarly, while MRI angiography offers superior soft-tissue contrast, it cannot dynamically assess lesion compressibility or vascular communications. For posterior mediastinal AVA, EUS demonstrates distinct advantages over these modalities. The 2023 *Medicina* review consolidates key diagnostic criteria, emphasizing EUS’s superiority in assessing mural thrombosis and compression effects on adjacent organs, which aligns with our dynamic EUS findings of an avascular cystic mass compressing the esophagus (20). EUS is one of the most effective dynamic examination methods for the diagnosis of mediastinal and abdominal vascular diseases, which can clearly understand the internal details and blood flow of the disease. There was a case in the literature where AVA was diagnosed by endobronchial ultrasound (EBUS) (21). EBUS is a tool for the assessment of mediastinal lesions close to the tracheobronchial tree. For posterior mediastinal lesions, EUS may have more advantages in their diagnosis and detailed assessment because esophageal EUS could be performed to understand the specific location, texture, specific size, blood vessels of the lesion, and its proximity to the esophagus, trachea, azygos vein, aorta, etc. Trojan reported that his team had once performed EUS and endoscopic ultrasound-guided fine-needle aspiration (EUS-FNA) on a case of posterior mediastinal cystic disease, which confirmed that the disease was an infected esophageal duplication cyst (22). There was also a case of acquired AVA caused by lung cancer confirmed by esophageal EUS (23). They demonstrated the impact of EUS and EUS-FNA for the management of posterior mediastinal cystic lesions in selected cases.

Therefore, transesophageal EUS with a loop scanning transducer (EU-ME2 GIF-UE 260; Olympus, Tokyo, Japan) was performed and confirmed that the lesion was an idiopathic anechoic lesion specifically located at the level of the middle of the esophagus from 25 to 29 cm to the incisor teeth. It was clearly distinct from the esophageal wall, seen as a classic fine-layer pattern, which appeared to compress the azygos vein and was close to the aorta. The maximum section of the lesion was 34.3 × 14.3 mm, the inner wall was smooth, no mural nodules were found, and an acoustic shadow could be seen. Finally, it was confirmed that the dysphagia was caused by the compression of the esophagus by mediastinal cystic lesions, which is not an enlarged lymph node, although not due to esophageal cancer or achalasia of the cardia, via EUS. In contrast to the very low complication rate associated with the use of EUS-FNA for the evaluation of solid masses (approximately 0.5%), perforation of cystic lesions, in general, harbors a 14% rate of non-fatal complications, mostly infection or hemorrhage (24). Therefore, EUS-FNA was not conducted further.

So far, there is no clear guideline or agreement on the treatment strategy for patients with AVAs. In the review of the existing data and from Maximilian’s clinical and scientific knowledge, interventional or surgical treatment should strongly be considered in cases with clinical symptoms, pulmonary embolism or pulmonary arterial hypertension, thrombus formation within the AVA in patients with oral anticoagulation, or for patients with contraindications to oral anticoagulants (25). Beyond symptomatic alleviation, surgical intervention for AVAs is fundamentally indicated for the prophylactic management of life-threatening complications. Contemporary evidence strongly supports resection in three high-risk scenarios: first, rupture prevention is warranted for saccular AVAs exceeding 3 cm in diameter, given their 12%–24% rupture risk attributable to wall stress concentration (16)—a critical consideration in our 3.5-cm case. Second, the presence of intralesional thrombus, pathologically confirmed in our patient (as demonstrated in **Figure 4B**), necessitates intervention due to an 8%–15% thromboembolic risk (11). Third, progressive organ compression by asymptomatic yet expanding aneurysms may cause irreversible esophageal or tracheal damage, as exemplified by our patient’s preoperative dysphagia.

Given these risks, thoracoscopic resection remains the definitive therapeutic standard, achieving near-complete symptom resolution and complication prevention in clinical series.

Compression of adjacent structures may significantly increase the risk of saccular AVA expansion, rupture, or thrombosis. There was a documented case of a patient with choking symptoms due to the esophagus being compressed by an AVA (11). In contrast, fusiform AVAs are asymptomatic and rather stable in long-term follow-up. Therefore, saccular AVAs, as in our case, also need surgical intervention. Thoracoscopic surgery is one of the most preferred treatment options for azygos vein aneurysm (9).

In order to avoid serious complications and relieve the patient’s obvious dysphagia caused by AVA at the same time, the cystic lesion in our case was surgically resected via a thoracoscopic approach. The diagnosis of AVA was made intraoperatively, as seen in **Figure 3**. After the operation, her dysphagia was also significantly relieved. It was confirmed later by postoperative pathology, as seen in **Figure 4**. H&E staining showed that clusters of thick-walled muscular vessels with irregular proliferation and expansion could be seen in the diseased fibrous adipose tissue, and red blood cells could be seen in the lumen, thus supporting the diagnosis of AVA rather than a lymph node or a cystic lesion.

Why did we initially misdiagnose this mass as a lymph node and a cystic lesion, but not an AVA, and why was there no blood flow signal in the lesion found in EUS? We should start from the cause of the azygos vein aneurysm. According to the etiology, AVAs were currently classified into idiopathic, acquired, or traumatic (16). Idiopathic AVAs may be related to embryonic development (15). The specific cause is unknown, and the incidence is extremely low. The causes of acquired AVA formation that have been proposed include portal hypertension, arteriovenous fistula, cardiac decompensation, pregnancy, cirrhosis, infection, or compression of the superior vena cava due to neoplasms or thrombus formation (16, 26). This patient had no history of heart failure, systemic hypervolemia, pulmonary hypertension, cirrhosis, infection, or chest injury. The combined imaging characteristics highlight that it is an idiopathic saccular AVA, which is a local expansion of the azygos vein and may form in the local wall of the azygos vein. The blood flow in the tumor was very slow, or thrombosis occurred; thus, it is similar to a solid tumor without obvious enhancement on CT. However, the lesion was cystic, and no blood flow signal was observed by EUS.

In conclusion, this case highlights that AVA is so far one of the rarest causes of dysphagia, which may present a point of esophageal obstruction. It is easily misdiagnosed as a mediastinal tumor or a lymph node, and its precise diagnosis is difficult to determine. EUS plays a very important and effective role in the vital preoperative evaluation of AVA. It is recommended for the differential diagnosis of AVA, while EUS-FAN is not recommended. Thoracoscopic surgical intervention can be performed to confirm the diagnosis and treatment of this isolated cystic AVA with clinical manifestations.

## Data Availability

The original contributions presented in the study are included in the article/supplementary material. Further inquiries can be directed to the corresponding author.
